# Premature Adult Death and Equity Impact of a Reduction of NO_2_, PM_10_, and PM_2.5_ Levels in Paris—A Health Impact Assessment Study Conducted at the Census Block Level

**DOI:** 10.3390/ijerph16010038

**Published:** 2018-12-24

**Authors:** Wahida Kihal-Talantikite, Pierre Legendre, Pauline Le Nouveau, Séverine Deguen

**Affiliations:** 1 LIVE UMR 7362 CNRS (Laboratoire Image Ville Environnement), University of Strasbourg, 67000 Strasbourg, France; 2 School of Public Health (EHESP), 35043 Rennes CEDEX, France; legendrepierre@LIVE.fr (P.L.); pauline.le-nouveau@eleve.ensai.fr (P.L.N.); severine.deguen@ehesp.fr (S.D.); 3 Sorbonne Universités, UPMC Univ Paris 06, INSERM, Institut Pierre Louis d’Epidémiologie et de Santé Publique (IPLESP UMRS 1136), 75646 Paris, France

**Keywords:** health impact assessments, premature death, equity impact, health impact, reduction of air pollution, environmental inequalities, spatial analysis, small area, AirQ

## Abstract

*Background*: To support environmental policies aiming to tackle air pollution, quantitative health impact assessments (HIAs) stand out as one of the best decision-making tools. However, no risk assessment studies have quantified or mapped the health and equity impact of air pollution reduction at a small spatial scale. *Objectives*: We developed a small-area analysis of the impact of air pollution on “premature” death among an adult population over 30 years of age to quantify and map the health and equity impact related to a reduction of air pollution. *Methods*: All-cause mortality data of an adult population (>30 years) from January 2004 to December 2009 were geocoded at the residential census block level in Paris. Each census block was assigned socioeconomic deprivation levels and annual average ambient concentrations of NO_2_, PM_10_, and PM_2.5_. HIAs were used to estimate, at a small-area level, the number of “premature” deaths associated with a hypothetical reduction of NO_2_, PM_10_, and PM_2.5_ exposure. In total, considering global dose response function for the three pollutants and socioeconomic deprivation specific dose response function, nine HIAs were performed for NO_2_ and six and four HIAs for PM10 and PM2.5, respectively. Finally, a clustering approach was used to quantify how the number of “premature” deaths could vary according to deprivation level. *Results*: The number of deaths attributable to NO_2_, PM_10_, and PM_2.5_ exposure were equal to 4301, 3209, and 2662 deaths, respectively. The most deprived census blocks always appeared as one of the groups most impacted by air pollution. Our findings showed that “premature” deaths attributable to NO_2_ were not randomly distributed over the study area, with a cluster of excess “premature” deaths located in the northeastern area of Paris. *Discussion*: This study showed the importance of stratifying an environmental burden of disease study on the socioeconomic level, in order to take into consideration the modifier effect of socioeconomic status on the air pollution-mortality relationship. In addition, we demonstrated the value of spatial analysis to guide decision-making. This shows the need for tools to support priority-setting and to guide policymakers in their choice of environmental initiatives that would maximize health gains and reduce social inequalities in health.

## 1. Introduction

Despite considerable improvement in prevention, management, and regulation, air pollution remains a leading environmental health issue worldwide. From a recent air quality model, the World Health Organization (WHO) estimates that 92% of the global population lives in places where air quality levels exceed WHO limits [1]. Air pollution has been identified as a health priority in the sustainable development agenda. Clean air is one of the fundamental requirements for human health and well-being [2].

While the increased risk of air pollution to health is relatively low compared to other risk factors, the total number of people affected is significant. According to the Organization for Economic Cooperation and Development [3], air pollution is known to be the main environmental cause of “premature” death. In 2012, WHO estimated from Global Health Observatory data that ambient air pollution contributed to 5.4% of all deaths worldwide [4]. However, while most studies have focused on estimating a relationship between pollution and health, less attention has been given to the differential health effects of air pollution according to the socioeconomic status, measured at individual and/or neighborhood levels [5,6]. Identifying population subgroups that are the most vulnerable to the effects of air pollution remains a public health research concern. Recent studies have suggested that several contextual or individual characteristics (such as gender and socioeconomic position, for example) could modify the association between exposure and mortality. Chen et al. in 2005 [7] found a significant increase of coronary death risk with PM_2.5_ exposure in women only, while Deguen et al. in 2015 [5] revealed a stronger association between short term variations of NO_2_ concentrations and all-cause mortality for subjects living in areas with low socioeconomic status.

Today, to support environmental policies aiming to tackle air pollution, quantitative health impact assessments (HIAs) stand out as one of the best decision-making tools, because they provide valuable information regarding the future health effects of a potential plan or policy. HIAs are already routinely used by the U.S. Environmental Protection Agency [8] in order to revise national ambient air quality standards. For instance, an increase in life expectancy of 0.61 years associated with a reduction of 10 μg/m^3^ in PM was estimated in the U.S. by Pope et al. in 2009 [9].

A study conducted in the Lausanne-Morges [10] urban area in Switzerland quantified the reduction in “premature” deaths due to air pollution reduction over a period of 10 years, and estimated a decrease of 1% to 2% of total all-cause annual deaths. In two French areas (the Grenoble and Lyon areas) [11], a recent study estimated at census block level that about 3–8% of deaths and 3–10% of lung cancer cases were attributable to PM_2.5_ exposure [11]. An HIA was also recently used to evaluate the health and economic impacts of a potential public transportation modification in terms of proposed fare increases and service cuts conducted in the U.S. state of Massachusetts [12]. To our knowledge, only a few epidemiological studies have investigated the health impact of reducing air pollution according to socioeconomic deprivation measured at a small spatial scale [13,14], ignoring within-city variations of air pollutants. In addition, in order to build efficient policies, it is crucial to establish a full and detailed socioeconomic and health-related assessment at the local scale and identify the categories of citizens who have multiple risk factors. However, no risk assessment studies have quantified or mapped the health impact of air pollution reduction at a small spatial scale to develop targeted policies, and more specifically, environmental policies. This study attempts to remedy this by developing a novel small-area approach combining an HIA and the clustering approach to map the health impact by socioeconomic deprivation level, and to investigate the equity impact of a reduction of ambient NO_2_, PM_10_, and PM_2.5_ concentrations.

In this context, this study has two objectives. First, we will estimate the number of “premature” deaths among an adult population older than 30 years associated with a reduction of NO_2_, PM_10_, and PM_2.5_ concentrations at the census block level in Paris, based on the counterfactual method [11]. Second, we will investigate the spatial distribution of the estimates number of “premature” deaths using a clustering approach to quantify how the number of “premature” deaths could vary according to neighborhood socioeconomic deprivation status measured at census block level.

## 2. Materiel and Methods

### 2.1. Study Area

The study area is the city of Paris (the capital of France). The population is about 2,250,000 inhabitants and about 1,360,000 inhabitants are over 30 years old. Paris is subdivided into 992 census blocks with a mean population of about 2199 inhabitants and a mean area of 0.11 km^2^.

### 2.2. Health Data

All-cause mortality data from January 2004 to December 2009 were considered and geocoded at the residential census block level in our study. The data were provided by the death registry of Paris. For confidentiality reasons, it was not possible to distinguish causes of mortality. According to the French demographic institute [15], the mortality rate is very low during childhood, then increases exponentially from age 30. In addition, causes of death for the population less than 30 years old are recognized to be mostly road injuries, domestic injuries, and suicide. For these reasons, and also because it was not possible to obtain the causes of death for reasons of confidentiality, we decided to exclude all the deaths of people aged under than 30 years [16]. The census block of residence was available for each case. In order to estimate death rate, we obtained the population size from the French National Census Bureau (INSEE: http://www.insee.fr). Ethical approval was obtained from the French commission on data privacy and public liberties (CNIL—Commission Nationale de l’Informatique et des Libertés, N 914118).

### 2.3. Air Pollution

Annual average ambient concentrations of NO_2_, PM_10_, and PM_2.5_ were modeled at census block level by the local air quality monitoring networks, corresponding to the Ile de France region for two different periods: from January 2004 to December 2009 for NO_2_ and from January 2007 to December 2009 for PM_10_ and PM_2.5_. The ESMERALDA Atmospheric Modeling system was used. This model integrates several data sources: meteorological data, linear emission sources, surface and major point sources, and background pollution measurements.

### 2.4. Socioeconomic Deprivation Index

To characterize the neighborhood socioeconomic deprivation at the census block level, an index was created in a previous study [17] (more details elsewhere by Lalloué et al. [17]). Briefly, Principal Component Analysis (PCA) was used to select 15 variables out of 41 initial socioeconomic and demographic variables provided by the 2006 national census at the census block level. Previous ecological studies have demonstrated this index’s ability to capture environment-related socio-spatial inequalities in France [6,18,19]. In order to capture the spatial variability of the pollutants, the socioeconomic index was categorized into 10 groups according to the decile of its distribution.

### 2.5. Health Impact Assessments (HIAs)

HIAs follow a methodology that requires diverse data sources. We combined information related to: (i) size of the population and level of their exposure (population exposure), (ii) the death rate in our study (baseline health rate), and (iii) dose-response function (the relative risk: RR).

The dose-response function was derived from epidemiological studies assessing the relative risk associated with the observed and/or modelled exposure [20]. In this study, the relative risk comes from WHO recommendations; the dose-response function relating all-cause mortality and long term NO_2_, PM_2.5_, and PM_10_ concentrations is quantified by RR = 1.041, 95% CI [1.019; 1.064], RR = 1.064, 95% CI [1.043; 1.085] and RR = 1.077, 95% CI [1.068; 1.086], respectively for 10 μg/m^3^ increase in exposure to the pollutant.

In our study, the health effects were evaluated for hypothetical air pollution reductions, according to WHO recommendations. The guideline values identified for each pollutant were 40 μg/m^3^ for NO_2_, 10 μg/m^3^ for PM_2.5_ and 20 μg/m^3^ for PM_10_.

The benefits of the air pollutant reduction scenarios are expressed in terms of attributable number of deaths per year (∆Y) estimated from the following equation:

(1) $$∆Y=Y0\times (1-e^{-\beta \times ∆x})$$

Where:

Y0 is the total number of observed deaths,

∆x is the difference between the yearly observed average of the air pollutant and the reference value (counterfactual), and

β is the natural logarithm of the dose-response function (the relative risk) expressed for a 10 μg/m^3^ increase in exposure to the air pollutant (β = ln(RR)/10).

The attributable number of deaths was estimated by AirQ+ software which was developed by the WHO European Centre for Environment and Health (http://www.euro.who.int/en/health-topics/environment-and-health/air-quality/activities/airq-software-tool-for-health-risk-assessment-of-air-pollution).

Table 1 and Table 2 present the input data required by the AirQ+ software for our two different periods: 2004 to 2009 for NO_2_ (Table 1) and 2007 to 2009 for PM_10_ and PM_2.5_ (Table 2).

To conduct an HIA per socioeconomic deprivation class, we used two studies which investigated the associations between all-cause mortality and long term air pollutant exposure by socioeconomic group: a Dutch study investigated NO_2_ and PM_10_ across 5 socioeconomic groups [14] and a Italian study investigated NO_2_ and PM_2.5_ across only 3 socioeconomic groups [13] (Table 3 and Table 4). Therefore, we estimated the attributable death rates separately for each socioeconomic class based on the 5 dose-response functions of the Dutch study and on the 3 dose-responses functions of the Italian study.

### 2.6. Spatial Analysis

The number of attributable deaths (estimated following the methodology described in Section 2.5) was distributed in each census block, proportionally to the adult population size living in the census block. To investigate the spatial distribution of “premature” deaths at census block level in Paris, we used a spatial scan statistic approach. The Poisson probability model used in the SaTScan software [21] was chosen as a cluster analysis method to detect the presence of high avoidable death spatial clusters (called ‘most likely clusters’).

The null hypothesis (H0) tested was that the risk is equi-probable throughout the study area. In other words, the expected “premature” death rate would be randomly distributed over the area. The alternative hypothesis (H1) was that there is an elevated risk within the cluster in comparison with census blocks outside the cluster. The procedure works as follows: a circle or window of variable radius (from 0 up to 50% of the population size as recommended by Kulldorf [22]) is placed at every centroid of the census block and moves across the whole study area. For each window, the “premature” death risk estimated in the window is compared with expected “premature” death rate under the hypothesis of a random distribution. The statistically significant most likely clusters are identified using the likelihood ratio test [23]. The *p*-value associated to each detected cluster was obtained from a Monte Carlo replication [24]. ArcGis software was used to map and visualize the spatial location of the statistically significant most likely clusters.

## 3. Results

### 3.1. Description of the Study Area and the Population

**Figure 1:**
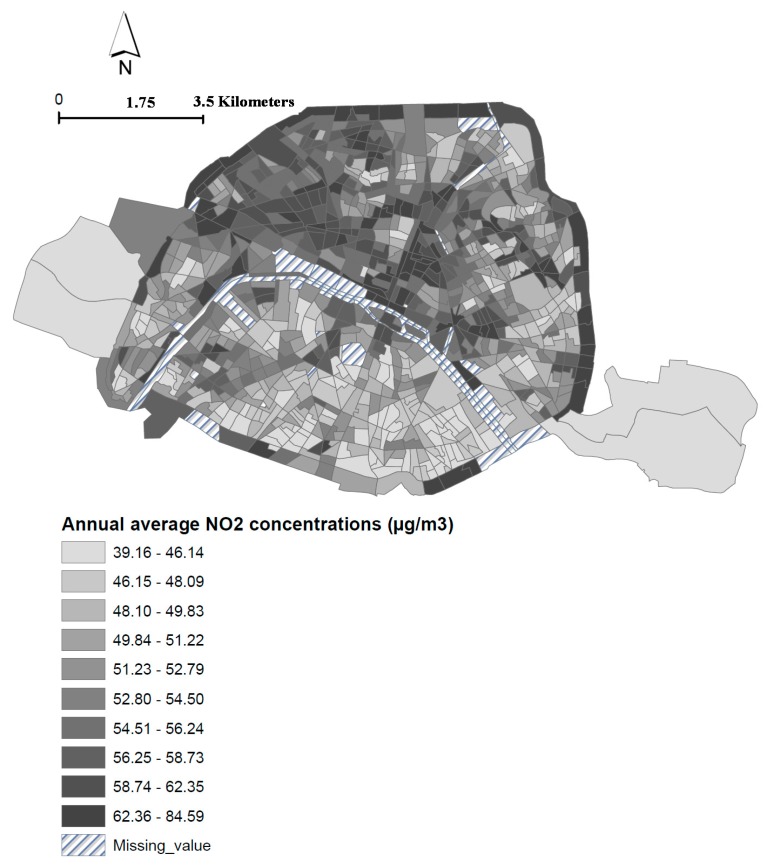
Spatial distribution of the annual average NO_2_ concentrations according to the deciles of its distribution, at the census block level, Paris city (Period: 2004–2009).

**Figure 2:**
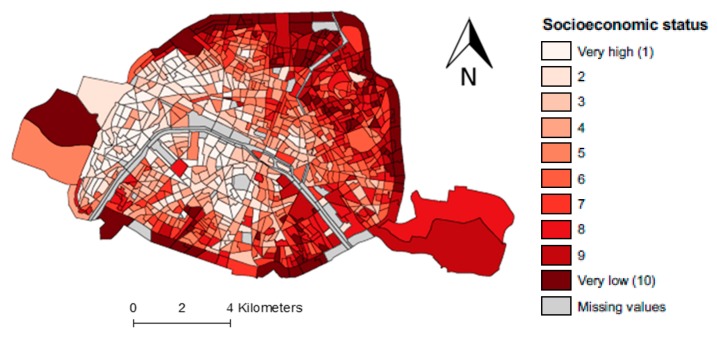
Spatial distribution of the socioeconomic deprivation index according to the decile of its distribution, at the census block level, Paris city.

**Figure A1:**
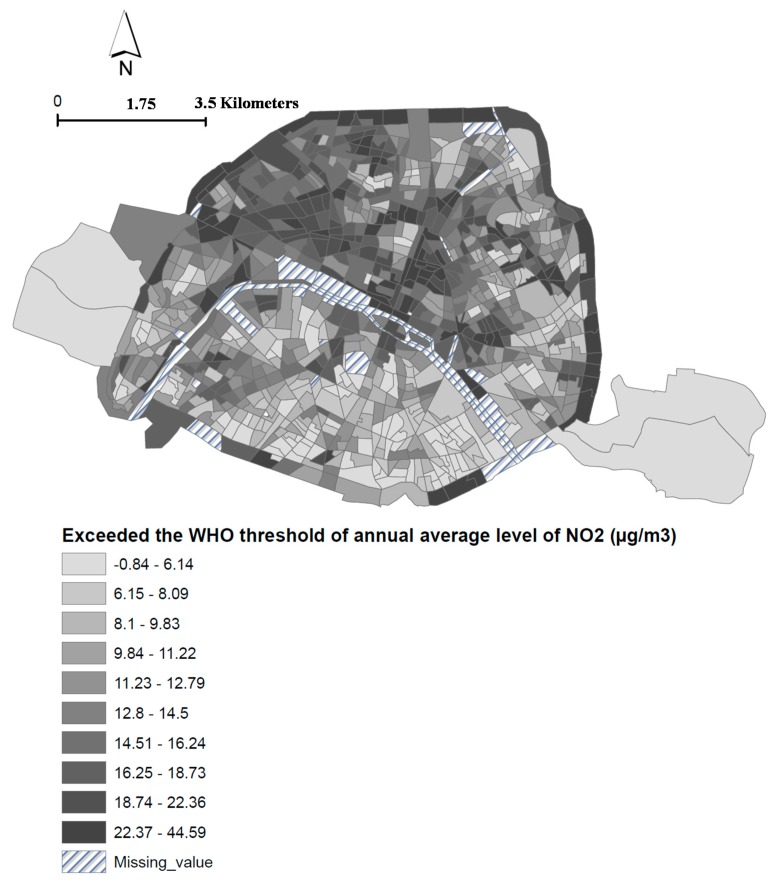
Spatial distribution of the exceeded the WHO threshold of annual average level of NO_2_ from 2000–2009 (the difference between level of NO_2_ exposure in each census block and WHO guidelines (40 µg/m^3^)).

**Figure A2:**
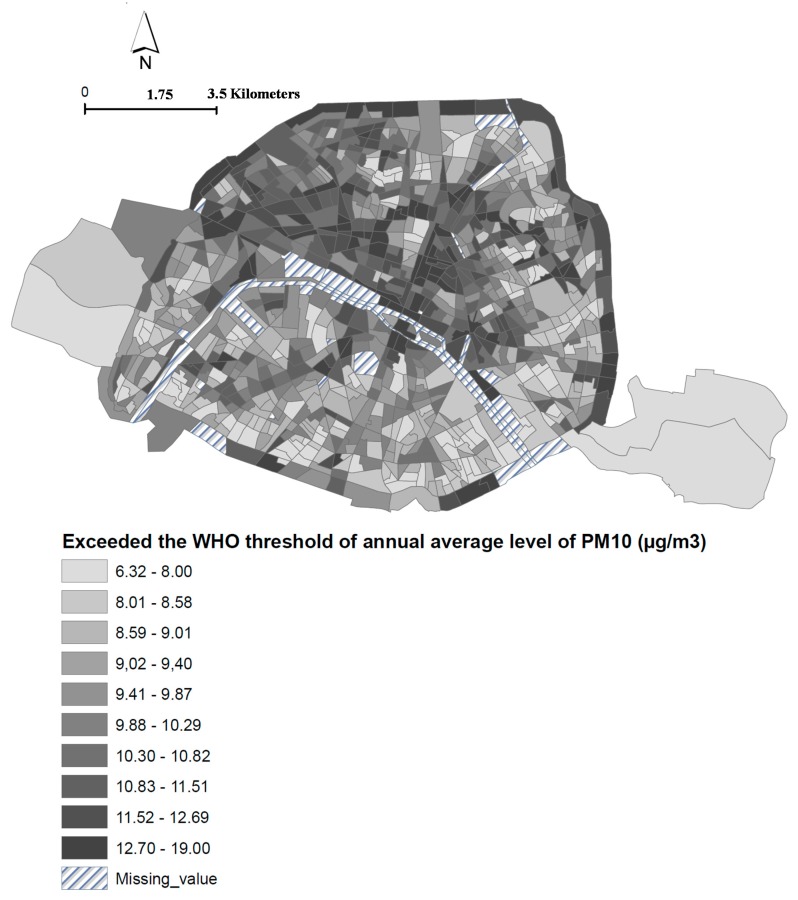
Spatial distribution of the exceeded the WHO threshold of annual average level of PM_10_ from 2000–2009 (the difference between level of PM_10_ exposure in each census block and WHO guidelines (20 µg/m^3^)).

**Figure A3:**
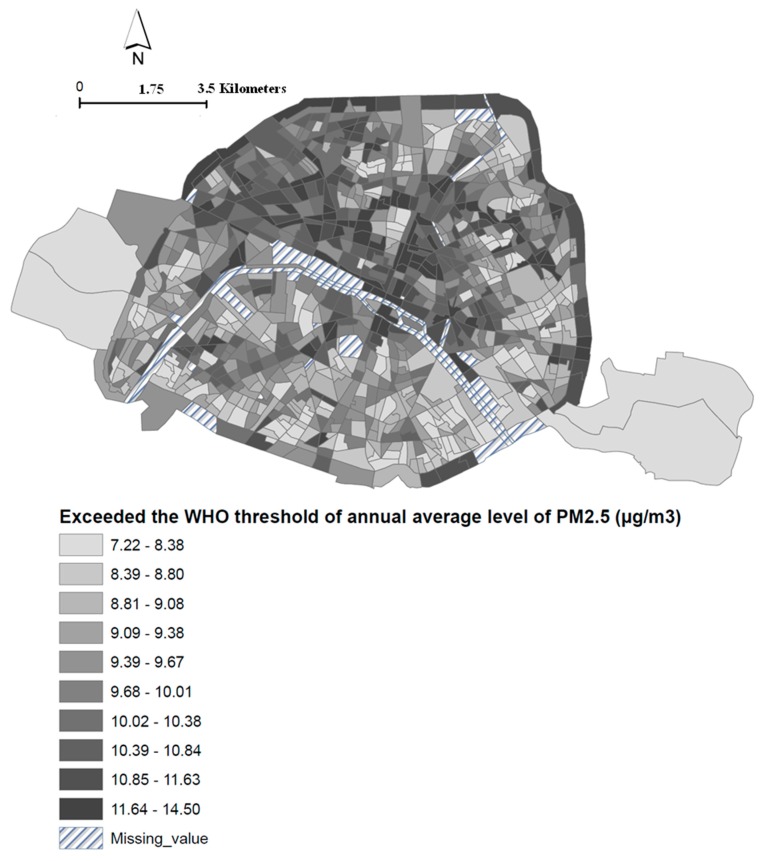
Spatial distribution of the exceeded the WHO threshold of annual average level of PM_2.5_ from 2000–2009 (the difference between level of PM_2.5_ exposure in each census block and WHO guidelines (10 µg/m^3^)).

A total of 82,117 deaths of people over 30 were registered in Paris between January 2004 and December 2009 (and 40,710 deaths between January 2007 and December 2009). The yearly average of NO_2_ concentrations was 53.4 μg/m^3^ (min = 38.6 μg/m^3^; max = 83.13 μg/m^3^). The yearly average of PM_10_ concentrations was 31.07μg/m^3^ (min = 24.4μg/m^3^; max = 43.3 μg/m^3^) and 20.9 μg/m^3^ (min = 14.9 μg/m^3^; max = 28.7 μg/m^3^) for PM_2.5_. The spatial distribution of the NO_2_ concentrations (Figure 1) shows a gradient from the south of the Seine River, with lower levels (the majority of the census blocks exhibit values less than 51 μg/m^3^), to the north, with higher levels (several of the census blocks, in dark colors, show values greater than 55 μg/m^3^). The spatial distributions of the annual average level of NO_2_, PM_10_ and PM_2.5_ exceeded the WHO threshold presented a similar pattern to the NO_2_ spatial distribution (see Appendix Figure A1, Figure A2 and Figure A3). The spatial distribution of the socioeconomic index (Figure 2) revealed a clear gradient from southwest (the least deprived areas), to northeast (the most deprived areas).

### 3.2. Health Impact

#### 3.2.1. Overall Estimates

Over the period 2004–2009, the number of deaths attributable to NO_2_ exposure was 4301 (95% CI [2044; 6545]), which corresponds to about 717 death per year (95% CI [340; 1091]) and to a rate of 52.8 (95% CI [25.1; 80.3]) per 100,000 inhabitants (aged > 30 years). It represents about 5% of total deaths among the adult population over 30 years of age.

For particulate matter, over the period 2007–2009, the number of attributable deaths was equal to 3209 (95% CI [1938; 3355]) and 2,662 (95% CI [2859; 3553]) for PM_10_ and PM_2.5_, respectively. This corresponds to a rate of 78.2 (95% CI [69.7; 86.6]) and 64.9 (95% CI [44.5; 84.4]) per 100,000 inhabitants (about 7.8% and 6.5% of total deaths for PM_10_ and PM_2.5_, respectively).

#### 3.2.2. Estimates by Socioeconomic Deprivation Class

Table 5 and Table 6 show the rate of attributable deaths estimated for the three air pollutants by decile of the socioeconomic deprivation index distribution.

Whatever the pollutant, the most deprived census blocks (decile 10) always appeared as one of the groups most impacted by air pollution. With an annual average of NO_2_ equal to 54.11 μg/m^3^ (one of the highest values), the attributable death rates are estimated to be 45.2 and 49.4 per 100,000 inhabitants using the dose-response function of the Dutch and Italian studies, respectively. With an annual average of PM_10_ and PM_2.5_ equal to 31.17 μg/m^3^ and 20.84 μg/m^3^ (in the high range of the annual average of air pollutants), the attributable death rates are estimated to be 100.1 and 54.0 per 100,000 inhabitants, using the dose-response function of the Dutch and Italian studies, respectively.

Populations living in less deprived census blocks (decile 3 and 4, in particular) also appear highly impacted by air pollution. These findings are consistent with the increase of the dose-response function and the level of air pollutant exposure in the high range, whatever the pollutant of interest.

### 3.3. Spatial Distribution

**Figure 3:**
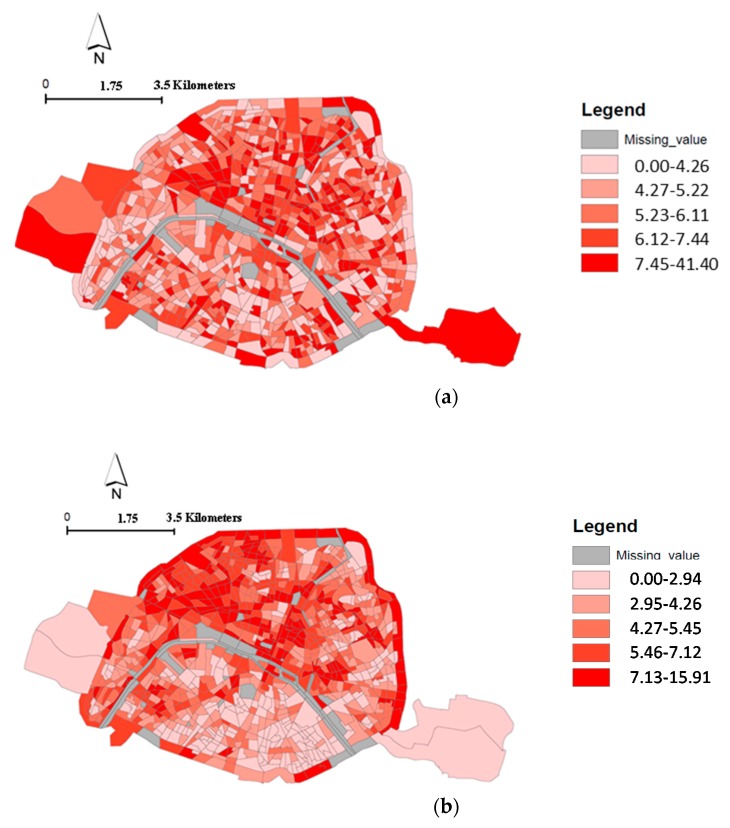

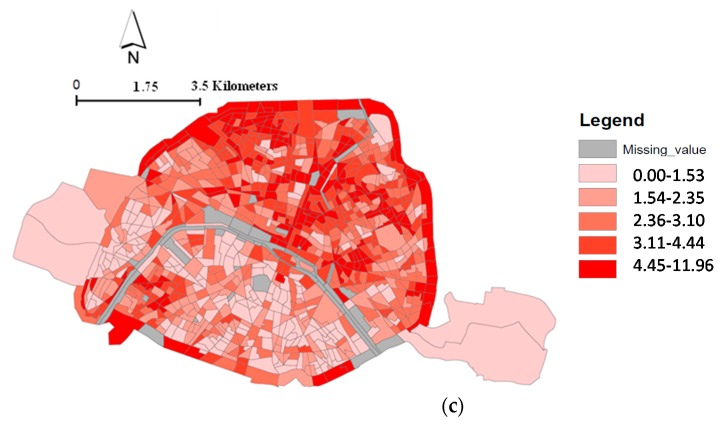
Spatial distribution of the rate of adults deaths attributable to NO_2_ among total death, at the census block level, Paris city; (**a**) without spatial variability of NO_2_ exposure in Paris; (**b**) with spatial variability of NO_2_ between census blocks; (**c**) with spatial variability of NO_2_ and socio-economic level between census blocks (according Dutch study [14]).

The rate of “premature” deaths per census block (Figure 3) was estimated according to 3 different scenarios: (a) without spatial variability of NO_2_ in Paris, (b) with spatial variability of NO_2_ between census blocks, and (c) with spatial variability of NO_2_ and socio-economic level between census blocks. The rate corresponds to the number of premature death divided by the number of total adult deaths.

Unlike the Figure 3a, the Figure 3b,c reveal a spatial pattern with a higher rate of “premature” deaths attributable to NO_2_ located in the north part of Paris in comparison with the south part. A difference also appears between Figure 3b,c: considering the spatial variability of NO_2_ combined with the level of socio-economic deprivation (based on Dutch study [14]), the higher rate of “premature” deaths among total adult death shifted in northeastern Paris (Figure 3c).

**Figure A4:**
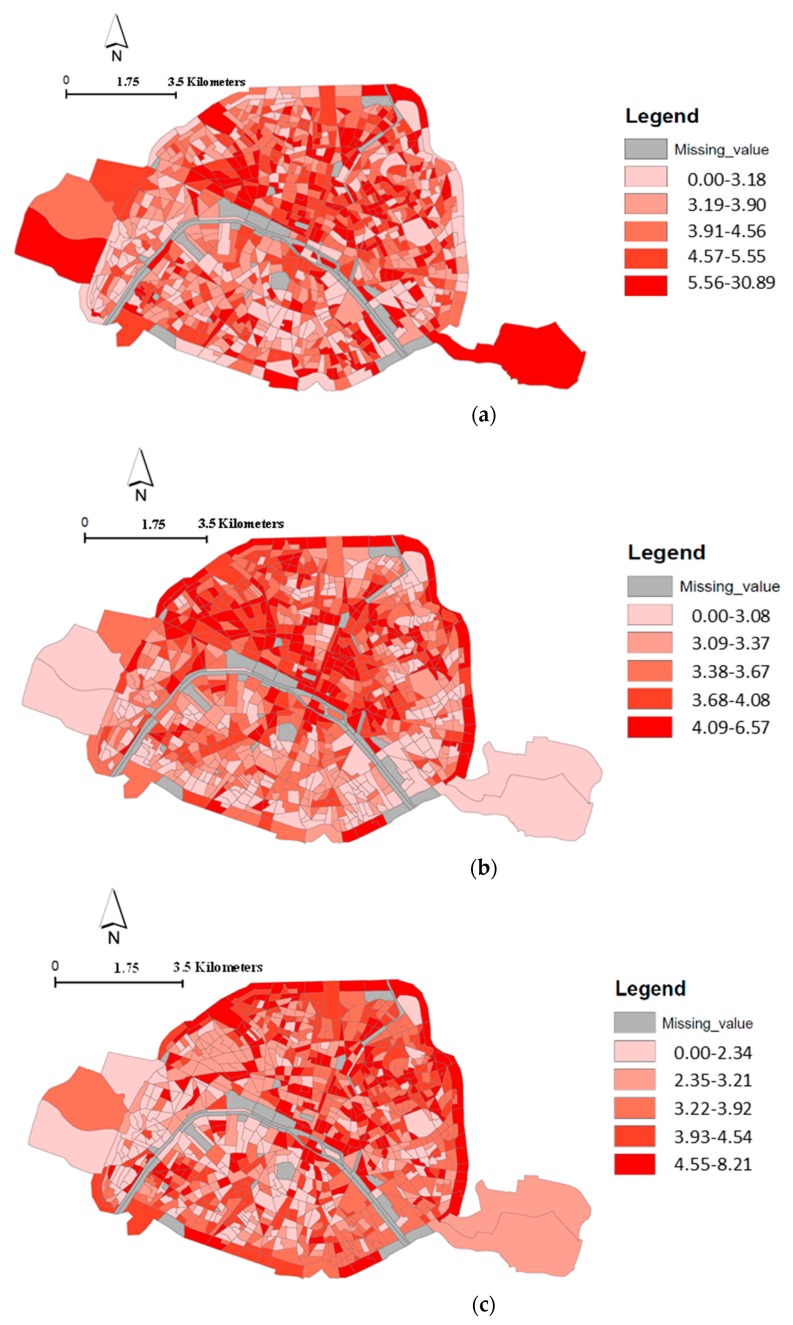
Spatial distribution of the rate of deaths attributable to PM_10_ among total death, at the census block level, Paris City; (**a**) without spatial variability of PM_10_ exposure in Paris; (**b**) with spatial variability of PM_10_ between census block; (**c**) with spatial variability of PM_10_ and socio-economic level between census block.

**Figure A5:**
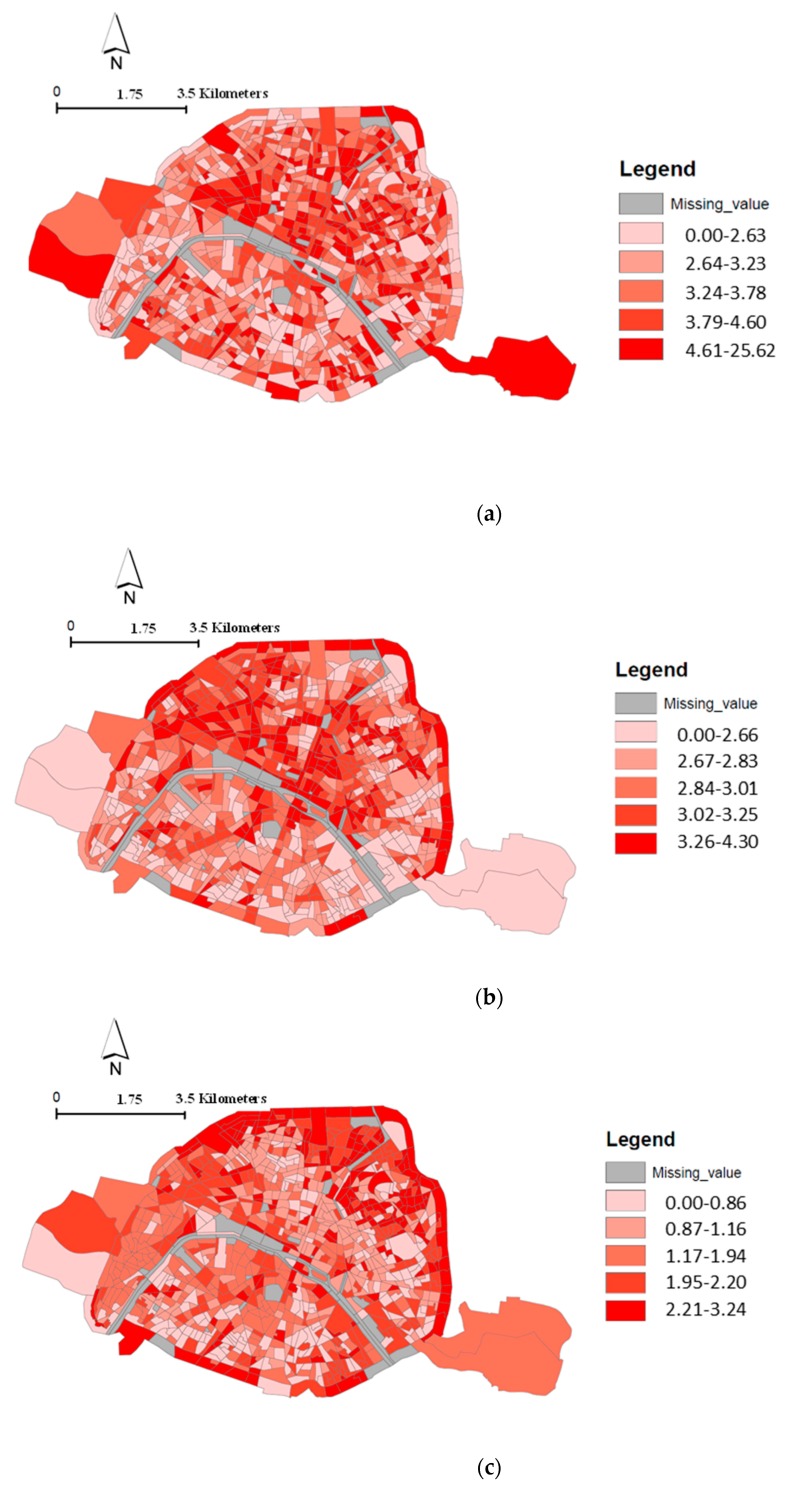
Spatial distribution of the rate of deaths attributable to PM_2.5_ among total death, at the census block level, Paris City; (**a**) without spatial variability of PM_2.5_ exposure in Paris; (**b**) with spatial variability of PM_2.5_ between census block; (**c**) tacking account the spatial variability of PM_2.5_ and socio-economic level between census block.

For particulate matter, the spatial distribution of the rate of the “premature” adult deaths attributable to PM_10_ and PM_2.5_, respectively, among total death, show the same pattern (see Appendix Figure A4 and Figure A5).

**Figure 4:**
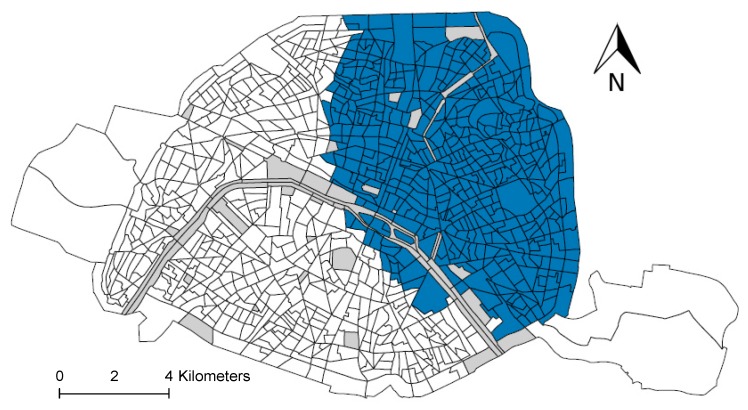
Mapping of the most likely cluster of the number of avoidable deaths. Blue areas identify census block included in the most likely cluster.

The statistical spatial approach confirms that the spatial aggregation of “premature” deaths in the northeast is significant (see Figure 4). This means that “premature” deaths are not randomly distributed across the study area. This most likely cluster comprises an area of 459 census blocks with a risk 1.12 times higher than in the rest of the study area (*p*-value  =  0.029). This cluster hosts a total of 4,038,108 inhabitants and has 3455 “premature” deaths (about 80% of the total number of “premature” deaths estimated in Paris). The spatial approach did not reveal any statistically significant aggregation of “premature” deaths attributable to PM_10_ and to PM_2.5_ (data not shown).

## 4. Discussion

In this study, we developed a small-area analysis of the impact of air pollution on “premature” death to quantify and map the health and equity impact related to a reduction of air pollution. We evaluated the health impact of hypothetical air pollution reductions according to WHO recommendations. This allowed us to estimate at a small-area level the rate of “premature” deaths attributable to NO_2_, PM_10_, and PM_2.5_ taking into account the level of socioeconomic deprivation, and to visualize the spatial distribution of the risk of “premature” deaths.

First, we predicted an overall mortality attributable to long-term NO_2_ exposure equal to 4301 deaths (5% of the total deaths registered in Paris over the period 2004 to 2009). Over the shorter period 2007–2009, the number of deaths attributable to PM_10_ and PM_2.5_ were comparatively higher: 3209 and 2662 deaths, which corresponds to about 7.8% and 6.5% of total deaths. This percentage was consistent with the Global Burden of Disease published in 2015 [25], which estimated that about 7.6% of total deaths were attributable to long-term exposure to PM_2.5_.

A recent study conducted in greater Cairo, Egypt estimated that about 11% and 8% of non-accidental mortality (in the population over 30 years old) could be attributed to PM_2.5_ and NO_2_, respectively [26]. The higher level of PM_2.5_ concentrations varying between 50 µg/m^3^ and 100 µg/m^3^ in this megacity may partially explain the difference observed with our estimate, the maximum concentrations of PM_2.5_ being equal to 28.7 µg/m^3^ in Paris. In contrast, because the NO_2_ concentration was found to be below the 40 µg/m^3^ air quality guideline of WHO, the author used another limit equal to 10 µg/m^3^, according to the recommendation of the Health Risks of Air Pollution in Europe project [27]. While in Paris the annual average NO_2_ concentration is higher, the stricter limit used in the Egyptian study may partially explain the difference with our estimate of deaths attributable to NO_2_. A study conducted in the Lausanne-Morges urban area of Switzerland estimated the health benefits of a reduction of PM_10_ and NO_2_ exposure after implementing a clean air plan [10]. Over a period of 10 years, the reduction of PM_10_ and NO_2_ exposure was equal to 3.3 μg/m^3^ and 5.6 μg/m^3^. These air quality improvements reduced total mortality by about 1% to 2%. Applying a similar reduction of PM_10_ and NO_2_ exposure in Paris produced comparable estimates of the percentage of “premature” deaths.

Second, our study demonstrated that the burden of mortality varied according to the level of socioeconomic deprivation. Populations living in the most deprived census blocks (those of the decile 10) appear particularly at risk of death related to NO_2_ exposure. Indeed, while the level of NO_2_ exposure decreases between the decile 5 and 9, population living in the census blocks of the decile 10 (the most deprived) accumulate a high level of exposure and a particular vulnerability to the adverse effect of air pollution. Consequently, for this population group, the two issues (exposure differential and vulnerability differential) may explain the high rate of death due to air pollution. However, it is not easy to draw a general statement about the most probable explanation between exposure differential, vulnerability differential, both because what we observed between socioeconomic level and NO_2_ exposure is not as clear with PM_10_ and PM_2.5_ exposure. Maybe, it could be partially explained by the lower spatial variability of PM.

Finally, our study showed that “premature” deaths attributable to NO_2_ were not randomly distributed over the study area, with a cluster of excess “premature” deaths located in the northeastern area of Paris.

To our knowledge, our study is the first to stratify an environmental burden of disease by the socioeconomic deprivation level measured at the residential census block level, making it difficult to compare our findings with those of others.

Several limitations of this study should be addressed here.

First, the methodology used to estimate attributable deaths is based on the AirQ+ software, which is based on a reference model developed by WHO. However, one weakness is that it does not take into consideration the effects caused by exposure to several pollutants in combination or their synergistic effects. In our study, as in the majority of scientific literature, the effects of pollutants are investigated individually, which could bias our estimates.

Secondly, the exposure level attributed to the population was approximated by the annual average ambient concentrations of the pollutants estimated at the place of residence provided at the date of death. This is a common limitation of numerous epidemiological studies which investigate the health impact of long-term exposure to air pollution, ignoring temporal and spatial variability due to mobility of the population and it could lead to a misclassification of the exposure. A conceptual model has been recently proposed aiming to assess cumulative exposure to air pollution at a fine scale and applied in Paris at the census block level [28]. The findings revealed that the level of population exposure to NO_2_ decreased when including the population mobility within the census block. However, the decrease was lower for the arrondissements located in northeastern Paris where the level of socioeconomic deprivation is the highest. This finding further supports the hypothesis of differential exposure.

Third, the socioeconomic deprivation status was estimated at the census block level rather than the individual level. However, census blocks are defined to maximize their uniformity in terms of population size, socioeconomic and demographic characteristics, land use, and zoning, thus reducing the risk of ecological bias.

Finally, the major limitation of our paper is the lack of studies that stratify their analysis based on socioeconomic deprivation status. Indeed, to produce a robust dose-response function per socioeconomic deprivation class, a meta-analysis is recommended. However, only two studies conducted in areas comparable to Paris were identified in the literature. Using the dose (air pollution)-response (mortality) function (relative risk) of these studies, our findings revealed that the number of “premature” deaths varied according to the socioeconomic deprivation level measured at the place of residence. This reflects not only the different dose-response functions used, but also the level of air pollution exposure and the population density. However, our findings tend to show a higher impact of air pollution exposure among the more deprived areas.

### Benefits of this Research for Public Health

This study provides answers to socioeconomic and environmental inequalities highlighted as an important public health issue by WHO. The research that formed the basis of public health policy provides little evidence for effective initiatives aiming to improve population health and tackle environmental and social inequalities in health. This paper is an attempt to fill the gap regarding the need for the development of powerful tools to support priority-setting and guide policymakers in their choice of environmental policies.

In this context, this study produced crucial information for policymakers to prioritize actions to investigate social health inequalities:

- Quantification of the number of “premature” deaths attributable to a reduction of NO_2_, PM_10_, and PM_2.5_ stratified by residential socioeconomic deprivation status.
- Spatial distribution of health and equity impacts of reducing these three pollutants.

In addition, this study illustrates the value of socio-spatial analysis implemented at a small spatial scale to pinpoint the areas where action is needed. In our study, for instance, we identified that an action conducted in northeastern Paris would be highly effective, since this area accounts for about 80% of the total number of “premature” deaths estimated.

At middle- and long-term, it could be really useful to perform the same study again with recent health and air pollution data, in order to investigate if the spatial distribution of the premature death changes over time, or if despite of the decrease of air pollution, cluster counting of a higher number of premature deaths related to air pollution is located in the same place.

## 5. Conclusions

This study showed the importance of stratifying an environmental burden of disease study on the socioeconomic level in order to take into consideration the modifier effect of socioeconomic status on the air pollution-mortality relationship. In addition, we demonstrated the value of spatial analysis to guide decision-making. Indeed, given today’s budgetary constraints, it can be quite challenging for policymakers to select an initiative. This shows the need for tools to support priority-setting and to guide policymakers in their choice of environmental initiatives that would maximize health gains and reduce social inequalities in health.

## Figures and Tables

**Table 1:** Descriptive statistics by socioeconomic deprivation class over the period 2004–2009. It corresponds to the input data of the health impact assessment (HIA) for NO_2_ exposure, only.

2004–2009	Population > 30 Years	Death Rate (per 100,000 Inhabitants)	NO_2_ (µg/m^3^)	Surface (km^2^)
Total	8,152,966	1007.20	53.39	105.4
1 (Less deprived)	718,290	1129.91	52.66	9.20
2	724,338	977.86	54.01	9.87
3	781,848	1006.33	54.10	7.54
4	802,248	985.23	53.53	7.14
5	782,280	1005.14	54.02	10.04
6	874,356	1002.57	53.73	6.66
7	872,640	917.90	52.63	7.17
8	862,818	1010.06	50.96	12.37
9	863,826	960.96	51.00	13.62
10 (More deprived)	819,006	1071.05	54.11	14.75

Death rate is the ratio between the total number of observed deaths older than 30 years and the total population older than 30 years. The death rate is expressed per 100,000 inhabitants. NO_2_ value corresponds to the mean of the annual average concentrations of the census blocks included in a given socioeconomic deprivation class. NO_2_: nitrogen dioxide. More precisely, values of NO_2_ are equal to $\frac{\sum_{i=1}^{N}\sum_{j=1}^{T}C_{ij}}{N*T}$, where N is the number of census block, T the number of years over the study period, and C the annual average concentrations of NO_2_ of a given census block (*i*) in a given year (*j*).

**Table 2:** Descriptive statistics by socioeconomic deprivation class over the period 2007–2009. It corresponds to the input data of the HIA for PM_10_ and PM_2.5_ exposure.

2007–2009	Population > 30 Years	Death Rate (per 100,000 Population)	PM_10_ (g/m^3^)	PM_2.5_ (g/m^3^)	Surface (km^2^)
Total	4,103,250	992.14	31.07	20.9	105.4
1 (Less deprived)	370,011	1112.67	30.8	20.74	9.20
2	370,800	924.22	31.24	21.01	9.87
3	395,459	979.87	31.34	21.07	7.54
4	407,266	982.4	31.16	21	7.14
5	394,149	987.19	31.31	21.09	10.04
6	442,374	964.57	31.15	20.96	6.66
7	443,739	901.21	30.9	20.83	7.17
8	435,338	986.82	30.29	20.41	12.37
9	442,427	933.94	30.34	20.42	13.62
10 (More deprived)	414,934	1048.12	31.17	20.84	14.75

Death rate is the ratio between the total number of observed deaths older than 30 years and the total population older than 30 years. The death rate is expressed per 100,000 inhabitants. PM_10_ (idem PM_2.5_ value corresponds to the mean of the annual average concentrations of the census blocks included in a given socioeconomic deprivation class. PM_10_: particulate matter 10 μm or less in diameter. PM_2.5_: particulate matter 2.5 μm or less in diameter. More precisely, values of PM_10_ (and PM_2.5_) are equal to $\frac{\sum_{i=1}^{N}\sum_{j=1}^{T}C_{ij}}{N*T}$, where N is the number of census block, T the number of years over the study period, and C the annual average concentrations of PM_10_ (PM_2.5_) of a given census block (*i*) in a given year (*j*).

**Table 3:** Associations between long-term NO_2_ and PM_10_ exposure and mortality all-causes by socioeconomic class extracted from the Dutch study [14].

Air Pollutants		Socioeconomic Deprivation Class				
		High (1–2)	Moderate/High (3–4)	Medium (5–6)	Moderate/ Low (7–8)	Low (9–10)
NO_2_	HR	1.013	1.036	1.022	1.027	1.031
	LL	1.002	1.027	1.014	1.02	1.025
	UL	1.023	1.046	1.03	1.034	1.038
PM_10_	HR	1.048	1.099	1.052	1.091	1.094
	LL	1.013	1.064	1.024	1.067	1.074
	UL	1.084	1.137	1.08	1.114	1.113

Socioeconomic deprivation class: decile; class ‘high’ = less deprived versus ‘low’ = more deprived; HR: Hazard Ratio; LL: lower limit of 95% confidence interval of the hazard ratio; UL: upper limit of 95% confidence interval of the hazard ratio; NO_2_: nitrogen dioxide; PM_10_: particulate matter 10 micrometers or less in diameter.

**Table 4:** Associations between long-term NO_2_ and PM_2.5_ exposure and mortality all-causes by socioeconomic class extracted from the Italian study [13].

Air Pollutants	Socioeconomic Deprivation Class			
		High (1–3)	Medium (4–7)	Low (8–10)
NO_2_	HR	1.024	1.016	1.034
	LL	1.012	1.002	1.024
	UL	1.036	1.03	1.045
PM_2.5_	HR	1.04	1.018	1.05
	LL	1.02	0.99	1.03
	UL	1.06	1.04	1.07

Socioeconomic deprivation class: decile; class ‘high’ = less deprived (decile 1, 2 and 3) versus ‘low’ = more deprived (decile 8, 9 and 10); HR: Hazard Ratio; LL: lower limit of 95% confidence interval of the hazard ratio; UL: upper limit of 95% confidence interval of the hazard ratio; NO_2_: nitrogen dioxide; PM_2.5_: particulate matter 2.5 μm or less in diameter.

**Table 5:** Rate of attributable deaths per socioeconomic class for two dose-response function.

Socioeconomic Deprivation Classes	Rate of Attributable Deaths (per 100,000 Population) [95% CI]	
	NO_2_ Dose-Response Function 1	NO_2_ Dose-Response Function 2
Decile 1 (less deprived)	18.3 [2.8; 21.1]	33.4 [16.9; 49.5]
Decile 2	17.5 [2.7; 30.7]	31.9 [16.2; 47.3]
Decile 3	48.9 [37.1; 61.8]	33.1 [16.8; 48.9]
Decile 4	46.0 [34.9; 56.2]	20.9 [2.7; 38.6]
Decile 5	30.2 [19.4; 40.8]	22.1 [2.8; 40.8]
Decile 6	29.5 [18.9; 39.9]	21.6 [2.7; 39.9]
Decile 7	30.4 [22.7; 37.9]	18.1 [2.3; 33.6]
Decile 8	33.2 [26.9; 40.4]	36.3 [25.9; 47.6]
Decile 9	31.7 [25.7; 38.6]	34.7 [25.7; 45.4]
Decile 10 (more deprived)	45.2 [36.7; 54.9]	49.4 [35.2; 64.5]

Dose response function 1 based on Dutch study [14] and dose response function 2 based on Italian study [13]. 95% CI: 95% Confidence Interval.

**Table 6:** Rate of attributable deaths per socioeconomic class for two dose-response function.

Socioeconomic Deprivation Classes	Rate of Attributable Deaths (per 100 000 Population) [95% CI]	
	PM_10_ Dose-Response Function 1	PM_2.5_ Dose-Response Function 2
Decile 1 (less deprived)	54.9 [15.4; 92.8]	45.9 [23.4; 67.5]
Decile 2	47.4 [13.3; 80.1]	39.1 [19.9; 57.4]
Decile 3	99.5 [66.5; 132.8]	41.6 [21.2; 61.2]
Decile 4	98.2 [65.7; 131.1]	19.1 [0; 41.5]
Decile 5	55 [26.1; 82.3]	19.3 [0; 42]
Decile 6	53 [25.1; 79.3]	18.7 [0; 40.6]
Decile 7	81.6 [61.5; 100]	17.2 [0; 37.5]
Decile 8	84.6 [63.7; 103.7]	48.9 [29.9; 67.1]
Decile 9	82.8 [66.5; 97.9]	46.3 [28.3; 63.6]
Decile 10 (more deprived)	100.1 [80.3; 118.1]	54.0 [33.3; 74.1]

Dose response function 1 based on Dutch study [14] and dose response function 2 based on Italian study [13]. 95% CI: 95% Confidence.
